# Insights into the Structure and Dynamics of Imidazolium Ionic Liquid and Tetraethylene Glycol Dimethyl Ether Cosolvent Mixtures: A Molecular Dynamics Approach

**DOI:** 10.3390/nano11102512

**Published:** 2021-09-27

**Authors:** Qianjin Guo, Qiang Liu, Yixin Zhao

**Affiliations:** 1 Academy of Artificial Intelligence, Beijing Institute of Petrochemical Technology, Beijing 100190, China; zhaoyixin@bipt.edu.cn; 2 Hydrogen Energy Research Center, Beijing Institute of Petrochemical Technology, Beijing 100190, China

**Keywords:** ionic liquids, hybrid binary mixtures, thermophysical properties, dynamical and transport properties, molecular dynamics (MD) simulations

## Abstract

In this work, the effect of molecular cosolvents tetraethylene glycol dimethyl ether (TEGDME) on the structure and versatile nature of mixtures of these compounds with imidazolium-based ionic liquid 1-butyl-3-methylimidazolium hexafluorophosphate ([bmim][PF_6_]) is analyzed and discussed at a molecular level by means of all-atom molecular dynamics (MD) simulations. In the whole concentration range of the binary mixtures, the structures and properties evolution was studied by means of systematic molecular dynamics simulations of the fraction of hydrogen bonds, the radial and spatial distribution functions for the various molecular ions and molecular species in the system, together with the snapshots visualization of equilibrated simulation boxes with a color-coding scheme and the rotational dynamics of coumarin 153 (C153) in the binary mixtures. The goal of the work is to provide a molecular-level understanding of significant improvement of ionic conductivity and self-diffusion with the presence of TEGDME as a cosolvent, which causes an enhancement to the ion translational motion and fluidity in the [bmim][PF_6_] ionic liquids (ILs). Under a mixture concentration change, the microstructure changes of [bmim][PF_6_] with the TEGDME molar fraction (X_TEG_) above 0.50 show a slight difference from that of neat [bmim][PF_6_] IL and concentrated [bmim][PF_6_]/TEGDME mixture in terms of the radial and spatial distribution functions. The relative diffusivities of solvent molecules to cations as a function of concentration were found to depend on the solvent but not on the anion. A TEGDME increase is found to be advantageous to the dissipation of the polar regions as well as the nonpolar regions in the [bmim][PF_6_] ionic liquids. These conclusions are consistent with the experimental results, which verified that the unique, complex, and versatile nature of [bmim][PF_6_]/TEGDME mixture can be correctly modeled and discussed at a molecular level using MD simulation data.

## 1. Introduction

In the past decade, room temperature ionic liquids have attracted extensive attention in academia and industry [1,2]. From various perspectives, especially in reaction, catalysis, and separation, their electrochemical properties have been widely used due to their unique physical and chemical properties, such as negligible vapor pressure, wide liquefaction range, high thermal/chemical stability and wide electrochemical window, etc. [1,2,3,4,5,6,7,8]. However, the use of pure ionic liquids is sometimes limited by high viscosity, high manufacturing cost, slow glassy dynamics, and difficult purification. An appropriate strategy to solve these problems and possibly adjust the performance of ionic liquids (ILs) is to mix them with simple, inexpensive, and low-weight molecular cosolvents. In this way, by properly selecting IL, cosolvent, and their appropriate molar fraction, the optimal design of such mixtures can be carried out, which not only leads to the decrease in viscosity but also generally expands various properties, thus opening up new application fields and wider operating conditions. Therefore, we need detailed information about the composition of mixtures and the target properties of such binary mixtures.

In recent years, a large number of studies have been carried out from both experimental and theoretical approaches to investigate the effects of water, alcohol, and other polar or nonpolar organic compounds in room temperature ionic liquids(RTILs). From an experimental point of view, nuclear magnetic resonance (NMR) and neutron/X-ray diffraction [9,10,11,12], vibration [13,14], fluorescence [15,16], and spectroscopy techniques have been widely used to investigate the behavior and the heterogeneity of IL/molecular liquid mixtures [17,18,19]. According to the research result, the appearance of hydrogen bond interaction between RTILs and cosolvent molecules can reduce the electrostatic attraction between cation and anion ions in ionic liquids, and then reduce the total binding energy of ionic liquids, thus reducing the viscosity [20,21,22,23,24]. Moreover, it has been observed that microviscosity was enhanced in the mixture of imidazolium-based ionic liquid 1-butyl-3-methylimidazolium hexafluorophosphate ([bmim][PF_6_]) and poly (ethylene glycol) (PEG) than that in the individual solvent. The hydrogen bond network formed by [bmim][PF_6_] with PEG is considered to be the main reason for the enhancement of microviscosity [25,26,27,28]. On the other hand, from the theoretical point of view, computer simulation plays an important role in RTILs’ molecular design due to their tremendous diversity. It can provide a detailed nanoscale characterization of the structure of ionic liquids in the liquid phase, which is not easy to obtain in experimental methods, and moreover, it allows the prediction of physicochemical properties [29,30,31,32,33]. Up to now, molecular simulation studies on RTILs have mainly focused on pure components and not provide a suitable picture about the relationship between ions molecular structure, intermolecular force, liquid phase structure, and macroscopic physicochemical properties. For this purpose, the development of systematic computational and experimental studies on the properties of ILs and organic solvents mixtures would provide the required information [34,35,36].

IL water system has been widely studied in cosolvent binary mixtures [32,33,34,35,36,37,38]. However, the exploration of binary mixtures of ionic liquids with other molecular solvents is quite limited [34,35,36,37,38,39,40,41,42]. In addition, ionic liquids are micro-heterogeneous in nature, so it is reasonable to expect that cosolvent can induce significant changes in their physicochemical properties [16]. It has been reported that the structure of pure ionic liquids is disturbed by various additives [43,44]. Therefore, it is very important to understand the structure organization and various intermolecular interactions for prevailing in IL-molecular solvent mixtures. Tetraethylene glycol dimethyl ether (TEGDME) can be used as a suitable cosolvent with [bmim][PF_6_] to study the interaction and properties of polar and nonpolar domains in such a hybrid system. In addition, unlike water or ethanol, TEGDME and [bmim][PF_6_] are completely miscible. Therefore, we can obtain the mixture of TEGDME and [bmim][PF_6_] with any mole fraction, which provides a wider range for the study of intermolecular interaction.

**Figure 1:**
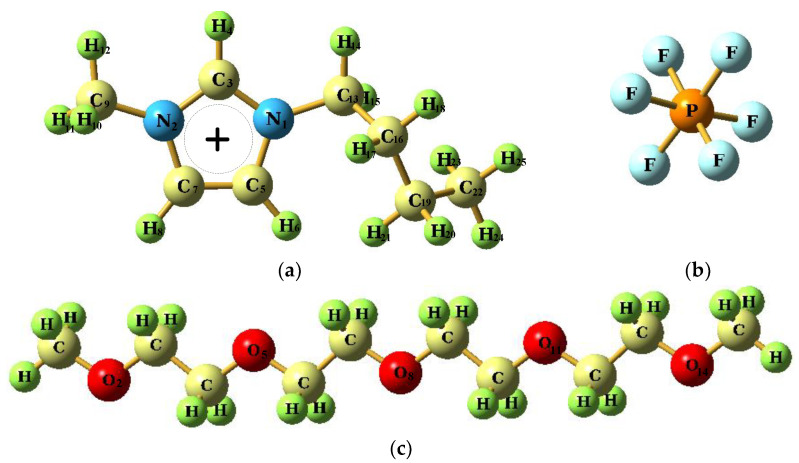
Chemical structures of (**a**) 1-butyl-3-methylimidazolium cation, [bmim]+, (**b**) hexafluorophosphate anion, ([PF_6_]^−^, and (**c**) Tetraethyleneglycol dimethyl ether(TEGDME).

In this work, the properties of [bmim][PF_6_]/TEGDME liquid mixtures were studied by using the theoretical method of classical molecular dynamics simulation (MD) in order to predict the thermophysical properties as a function of the composition of the mixture, characterize the liquid structure at the nanoscopic level, and infer the relationship between the microstructure and the macroscopic properties. The MD simulations were used to reveal the microscopic molecular details of the dynamics, transport properties, and structure, and mechanical properties of the ionic species in such a hybrid system of [bmim][PF_6_] with TEGDME (Seen in Figure 1). For this reason, we use atomic and molecular dynamics simulation as an appropriate microscopic tool to calculate various quantities and discuss the influence of adding different mole fractions of TEGDME cosolvent molecules on the fine-tuning of different properties of [bmim][PF_6_] IL, as well as the influence of the gradual increase in the cosolvent on the ionic state and nano-segregation of the solution.

## 2. Materials and Methods

### 2.1. Force Field Parameters

The force field used in this work for the ILs is from the systematic all-atom force field developed by Canongia Lopes et al. [42,43,44] based on the optimized potentials for liquid simulations (OPLS) and Assisted Model Building and Energy Refinement (AMBER) framework with some minor modifications as shown in Equation (1).

(1) $$V_{total}=\underset{nbonds}{\sum }\frac{k_{b}}{2}{\left(r-r_{eq}\right)}^{2}+\underset{angles}{\sum }\frac{k_{\theta }}{2}{\left(\theta -\theta_{eq}\right)}^{2}+\underset{dihedrals}{\sum }\frac{k_{\varphi }}{2}{\left[1+\cos\left(n\varphi -\delta \right)\right]}^{}+\underset{i<j}{\sum }4\varepsilon_{ij}{\left[{\left(\frac{\sigma_{ij}}{r_{ij}}\right)}^{12}-{\left(\frac{\sigma_{ij}}{r_{ij}}\right)}^{6}\right]}^{}+\underset{i<j}{\sum }\left(\frac{q_{i}q_{j}}{4\pi \varepsilon_{0}r_{ij}}\right)$$

where *V_total_* is the total energy of the system, which is related to dihedral angle, bond length, bond angle, electrostatic interactions, and van der Waals (VDW) interactions. The nonbonded interactions comprise Lennard-Jones and Coulombic potential terms. The force constants for the bonded interactions are expressed as *k*_b_, *k*_θ_, and *k*_ψ_. The dihedral angle bond length and bond angle for the equilibrium structure is expressed as *d*, *r*_eq_, and *q*_eq_, respectively. *q*_i_ is the charge of atom *i*, and *r*_ij_ is the distance between atoms *i* and *j*. *ε*_ij_ and *d*_ij_ are the Lennard-Jones (LJ) parameters for different atoms.

The force field parameters of C153 molecules are carried out using the fully atomistic force field, which originates from the OPLS-AA methodology [45,46,47,48,49]. The TEGDME molecules were represented using the OPLS-UA model proposed by Jorgensen and coworkers [50].

### 2.2. Simulation Details

Molecular dynamics simulations were performed with GROMACS 4.6.1 software package [51]. Simulations consisted of a single C153 centered in a cubic box containing (1 − *x*) [bmim][PF_6_] + *x* TEGDME binary mixtures where *x* stands for TEGDME mole fraction, covering the whole composition range in 0.1 steps were built for the number of ions and molecules reported in Table 1. These initial configurations were constructed in large cubic boxes to provide systems of low density, which were then allowed to relax in order to contract the cell to provide the desired densities. This was achieved by a series of low-temperature constant-pressure constant-volume simulations with time steps up to 3 orders of magnitude smaller than that used in production runs. Initially, these cations, anions, and consolvent molecules were randomly scattered in a low-density cubic box with periodic boundary conditions. After the energy minimization using the steepest descent method, the volume of the box was compressed to a normal value by performing a 500 ps simulation in the NPT ensemble. The resulting configuration was used for the subsequent equilibrium run. To obtain a better equilibrium structure, our simulations in the equilibrium stage were carried out by using the Berendsen thermostat/barostat algorithm at a series of decreased temperatures: 600, 500, and 400 K. In each case, the trajectory was run for a duration of 2 ns, and the final configuration obtained was used as the starting point for the next simulation at the subsequent lower temperature.

After equilibration, at the set target temperature, all simulations were carried out in the isobaric-isothermal (NPT) ensemble with a pressure of 1 bar and compressibility of 4.5 × 10^−5^ bar^−1^. A leapfrog algorithm with a time step of 1 fs was used to integrate Newton’s equations of motion. The cutoff distance of the neighbor searching was set to 1.2 nm. The switching function, which started at 0.9 nm, was used to smoothly truncate the van der Waals potential at the cutoff distance of 1 nm, and long-range dispersion corrections were applied for energy and pressure. The linear constraint solver algorithm was used for all bonds involving H atoms. Each trajectory was evaluated using the program package Trajectory Analyzer and Visualizer(TRAVIS) [52]. The 2- and 3-dimensional functions were generated with Visual Molecular Dynamics(VMD) [53].

Simulations of the rotational dynamics of C153 in [bmim][PF_6_] and TEGDME mixtures at 273, 300, 313, and 353 K were also carried out using the fully atomistic force field described above. At each temperature, pre-equilibration with charge scaling (0–100%) and Constant temperature, constant volume (NPT) equilibration at 1 bar were followed by 5 ns simulations in the Constant energy, constant volume (NVE) ensemble using the velocity Verlet algorithm with a 1 fs time step. Nonbonded interactions were calculated using the same parameters previously described for the case of TEGDME in [bmim][PF_6_]. Liquid structure, diffusion, and conductivity were based on 10 ns simulations (after 5 ns of equilibration) with all atomic positions saved every 1 ps.

## 3. Results

The MD study reported in this work has two main purposes. First, the most relevant thermophysical properties of [bmim][PF_6_] + TEGDME mixture are predicted as a function of the composition of the mixture and compared with the available experimental information. In this way, the physical model (force field parameterization) used to describe the involved molecules is verified, and the applicability of the proposed MD method to describe the macroscopic properties of these systems is analyzed. Moreover, the calculations of these physicochemical characterizations allow inferring the thermodynamic properties of the mixed system from the predicted ideal deviation. Second, the results of molecular dynamics allow a detailed description of intermolecular forces and the spatial arrangement of molecules. Therefore, with these two purposes, MD results are discussed in the following sections.

### 3.1. Density for Evaluation of Forcefields

The force field can be verified by some characteristics, such as density, spectral data, and heat of vaporization [53,54,55,56,57]. Among them, density is one of the most basic properties of RTILs, and it is also one of the most accurate experimental data sources, which can be directly obtained from isothermal−isobaric molecular dynamics simulation. Therefore, density is usually the most common property used to verify the force field parameters in monocationic ionic liquids (MILs) and dicationic ionic liquids (DILs), because the calculation of the density is simple, and very accurate experimental values can be obtained [55]. Generally, it is expected that the excellent predictive capabilities could be observed with the developed force field, and the difference between the predicted value and the experimentally measured value is less than 1%. In this work, The density of [bmim][PF_6_] and TEGDME were calculated to be 1.357 g/cm^3^ and 1.018 g/cm^3^ at 298 K and 0.1 MPa, respectively. The experimental values are 1.366 g/cm^3^ and 1.009 g/cm^3^ [58,59,60,61]. The error is 0.65% and 0.98%. Therefore, the proposed force fields for [bmim][PF_6_] and TEGDME are reliable.

### 3.2. Structural Analysis

**Figure 2:**
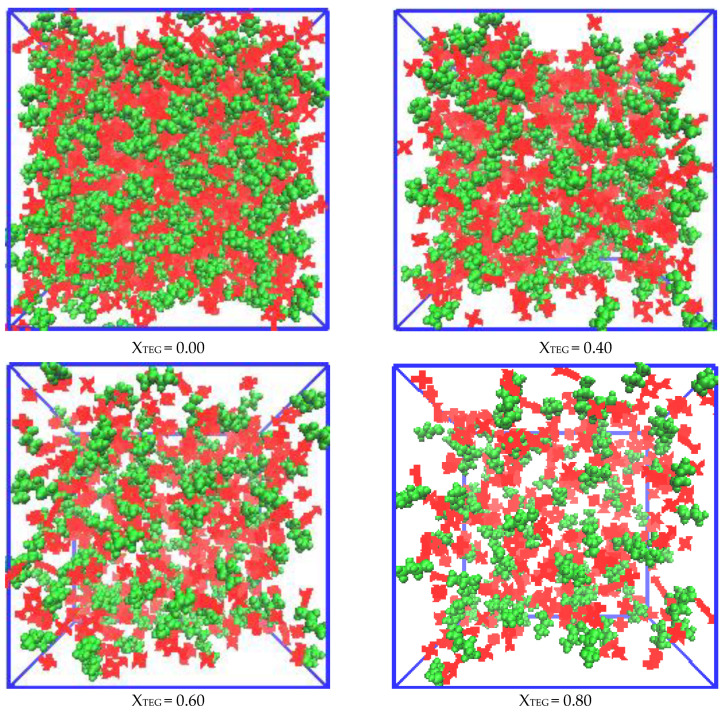
Simulation snapshots of the investigated [bmim][PF_6_] IL/TEGDME mixtures. The snapshots of equilibrated simulation boxes are slices with size 50 × 50 × 50 Å and refer to the mixture with X_TEG_ = 0.00, 0.40, 0.60, and 0.80 at 300 K. The polar domains that include imidazolium rings with methyl side chains of cations coded with the red color is coded with the red color scheme. The anions and nonpolar regions that contain hexyl side chains of the cations are coded with a green color scheme.

**Figure 3:**
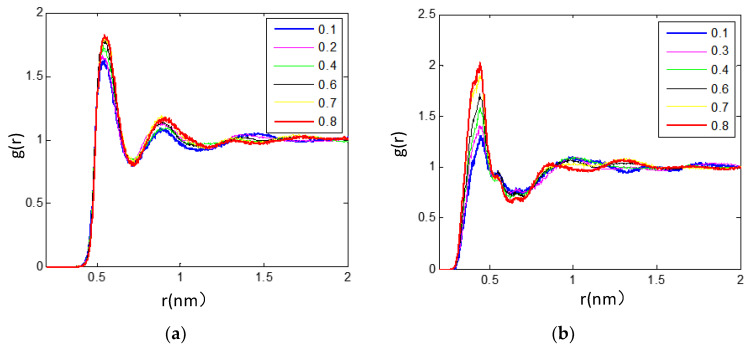
RDFs between the (**a**) (O_8_–P) RDFs for anion-TEGDME pairs, (**b**) cation-TEGDME neighbors to investigate the influence of adding TEGDME on the various structural correlations between the species in the concentration range of 0 ≤ X_TEG_ ≤ 1 at 300 K.

**Figure 4:**
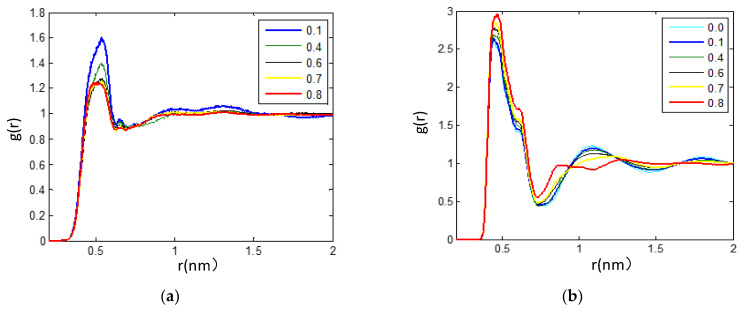
RDFs between the (**a**) (O_8_–O_8_) RDFs for TEGDME–TEGDME (**b**) (ring-P) RDFs for cation–anion neighbors to investigate the influence of adding TEGDME on the various structural correlations between the species in the concentration range of 0 ≤ X_TEG_ ≤ 1 at 300 K.

**Figure 5:**
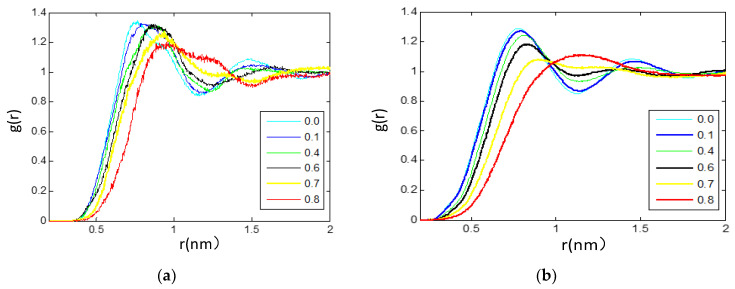
RDFs between the (**a**) (ring–ring) RDFs for cation–cation pairs, (**b**) (P–P) RDFs for anion–anion pairs, and neighbors to investigate the influence of adding TEGDME on the various structural correlations between the species in the concentration range of 0 ≤ X_TEG_ ≤ 1 at 300 K.

**Figure 6:**
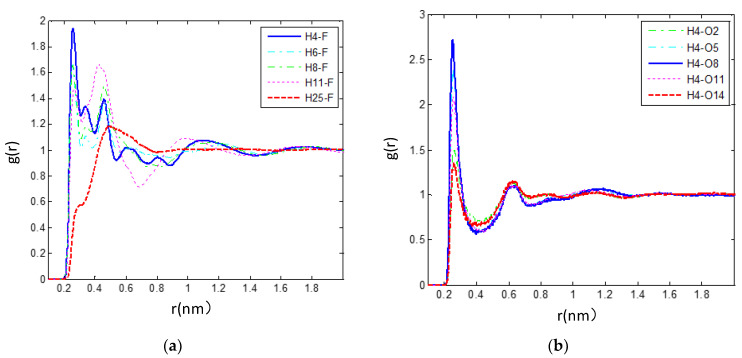
(**a**) Comparison of the RDFs between the fluorine atom (F_1_) in the anions and selected hydrogen atoms of the cations (H_4_, H_6_, H_8_, H_11_, H_14_). This is evidence of strong hydrogen bonding between the H_4_ atom of the cations and the fluorine atom (F) of [PF_6_]^-^ anions. (**b**) Calculated RDFs between the hydrogen atom (H_4_) of the cations and selected oxygen atoms of the TEGDME molecules (O_2_, O_5_, O_8_, O_11_, O_14_) for X_TEG_ = 0.6 from MD simulations at 300 K. It seems that the strong hydrogen bonding occurs between the oxygen atom (O_8_) of the TEGDME molecules and the head group (imidazolium ring) of the cations.

**Figure 7:**
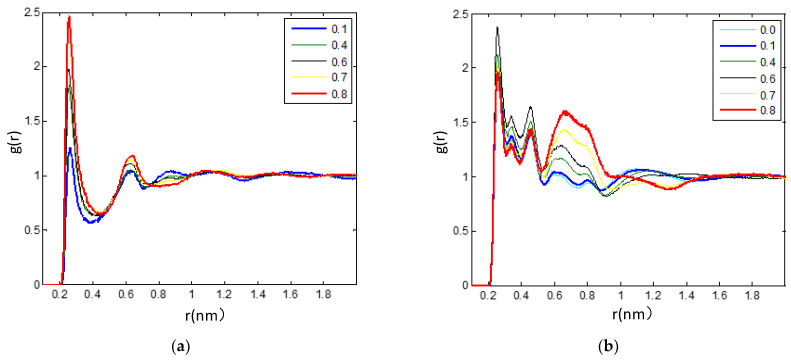
(**a**) (H_4_-O_8_) RDF for description of the H-bond between the IL anion and the TEGDME with the different TEGDME mixtures at 300 K. (**b**) (H_4_-F) RDF was carried out to study the H-bond details between the cations and anions under the different TEGDME mixtures at 300 K in this work.

**Figure 8:**
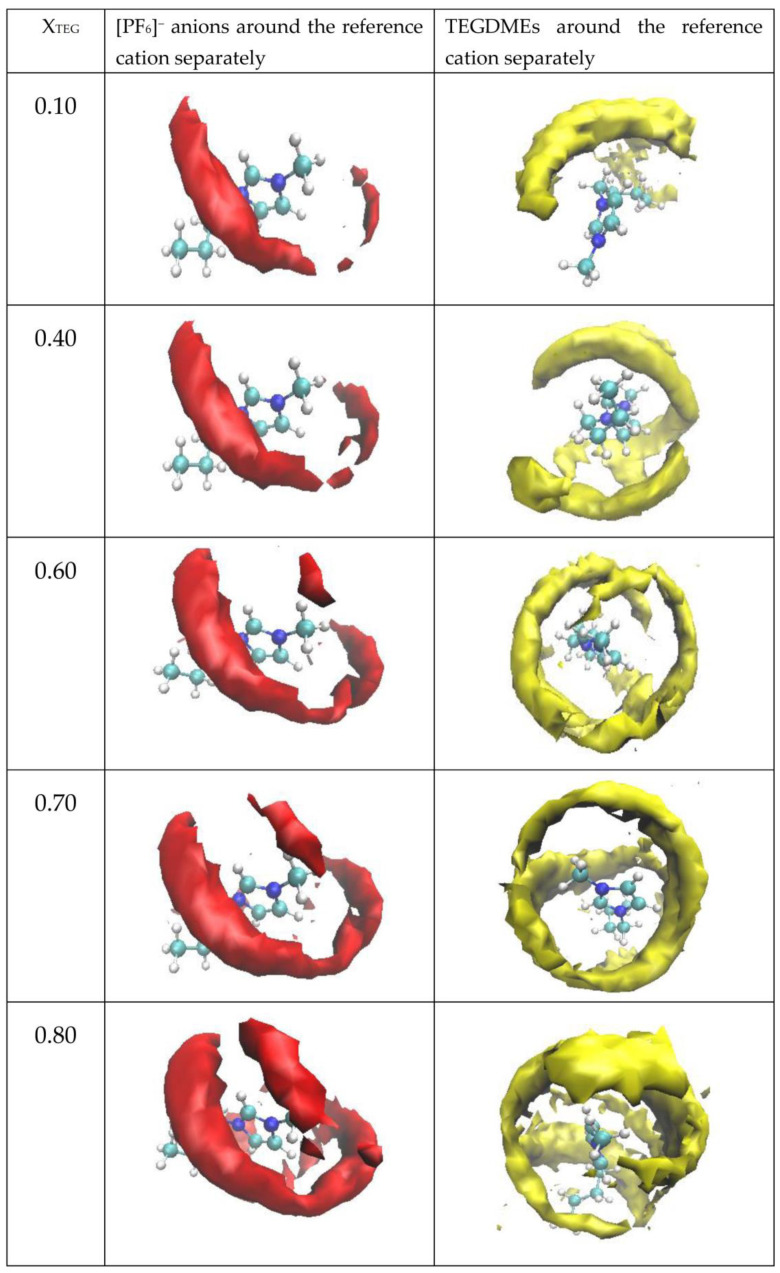
Calculated SDFs of the center of masses of [PF_6_]^−^ (red density clouds) around [bmim]+ and TEGDME (yellow density clouds) around [bmim]+ as the reference cation in the case of X_TEG_ = 0.10, 0.40, 0.60, 0.70, and 0.80 at 300 K. First column panels represent [PF_6_]^−^ anion’s probability around the reference cation separately and, whereas the last column shows the TEGDME density clouds around the reference cation at the different molar fraction of TEGDME.

**Figure 9:**
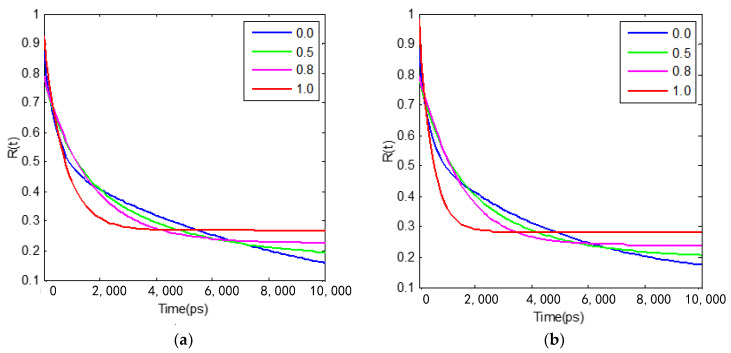
Theoretical rotational dynamics of C153 in [bmim][PF_6_] and TEGDME mixtures with the mole fractions (X_TEG_) of TEGDME of 0 ([bmim][PF_6_]), 0.5, 0.8 and 1 (TEGDME), respectively. (**a**) is the fitting result at 300 K 1 atm, (**b**) is the fitting result at 353 K 1atm.

**Figure 10:**
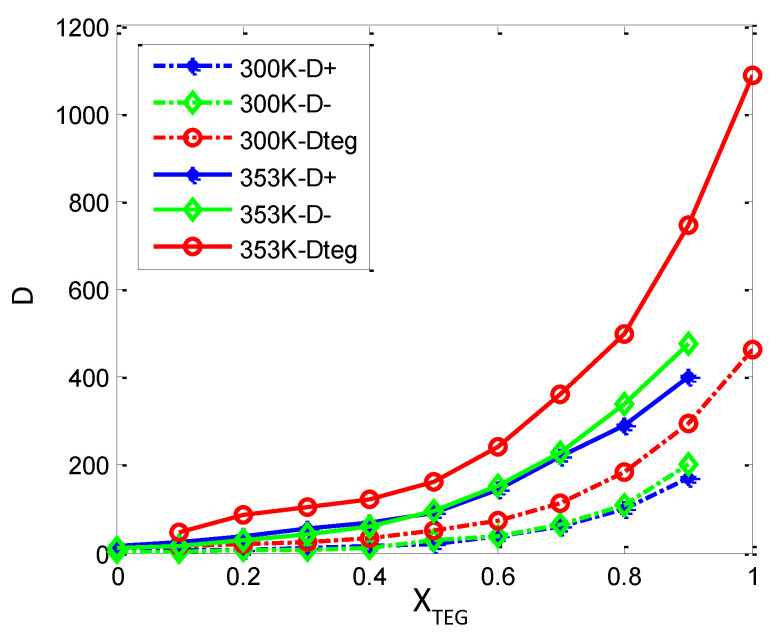
Self-diffusion coefficients, D+, D−, Dteg, for (1−x) [bmim][PF_6_] + x TEGDME mixtures obtained from molecular dynamics simulations in this work, MD, at 300 K and 1 atm. All D values from MD at 353 K and 1 atm from mean square displacements (MSD) and Einstein’s equation with β = 1 for all the mixtures. β stands for the slope of MSD vs. time log−log plots. D+ stands for cation, D− for anion, and D_teg_ for TEGDME.

**Figure 11:**
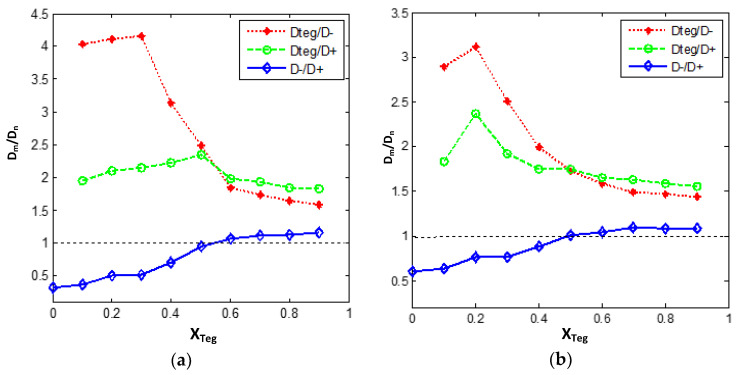
Self-diffusion coefficients for [bmim]+, [PF_6_]^−^, and TEGDME molecule in [bmim][PF_6_]/TEGDME mixtures as a function of the TEGDME molar fraction (**a**) at 300 K (**b**) at 353 K. At X_TEG_ > 0.50, the anions diffuse faster than the cations in the solution.

In order to further explore the species structure arrangement and nanoscale structural heterogeneity of [bmim][PF_6_] IL and its binary mixture with different concentrations of TEGDME, this section calculates the partial site-site radial distribution function (RDF), analyzes the snapshot visualization of equilibrium simulation box by the color-coding scheme, and the spatial distribution function (SDF) simulated by MD at 300 K. The results are shown in Figure 2, Figure 3, Figure 4, Figure 5, Figure 6, Figure 7, Figure 8, Figure 9, Figure 10 and Figure 11.

#### 3.2.1. Morphologies of Microscopic Structure in IL/TEGDME Mixtures

According to previous experiments and simulations, the microscopic structure of pure [bmim][PF_6_] IL can be separated by polar and nonpolar domains at the nanoscale [16]. The polar domain for pure [bmim][PF_6_] IL is composed of [PF_6_]^−^ and [bmim]^+^ imidazole rings, which contains the highest density of cationic charges because of electrostatic interaction. On the other side, the nonpolar domain is formed by the aggregation of alkyl side chains, which is attributed to the interaction between the alkyl side chains of cations due to the attractive van der Waals interaction. Here, a series of snapshots from the simulation frame is provided to more intuitively insight into the nature and evolution of the microscopic structure with the gradual adding TEGDME into [bmim][PF_6_] ILs. To show the phenomenon of evolution in the microscopic structure clearly, a color-coding scheme is used for visualization of polar and nonpolar segregation in [bmim][PF_6_] IL at 300 K. A simulated snapshot depicted according to the red/green polar/nonpolar convention is shown in Figure 2, where the polar portion includes an imidazoline ring with a cationic methyl side chain and an anionic as well as a nonpolar region with a cationic hexyl side chain. From Figure 2 of pure ionic liquids (X_Teg_ = 0.0), it can be seen that the nonpolar domain is separated from the polar network, which includes imidazole rings and anions with high charge density. When the mole fraction X_Teg_ of TEGDME increases from 0.10 to 0.80 (Figure 5), the ion aggregations will be dissociated until the size of the ion clusters is effectively reduced and transformed into the thinner dispersed part of TEGDME molecules, which can be attributed to the gradual weakening of the interaction between ions till X_Teg_ = 0.80.

Simulated snapshots provide a powerful visual insight into the nature and evolution of metastable structures as TEGDME increases. However, if the radial distribution function (RDF) is considered, a more quantitative analysis can be carried out. Therefore, we will discuss the radial distribution function analysis of microscopic ionic structure in the next section.

#### 3.2.2. Radial Distribution Functions Analysis of Microscopic Ionic Structure

Another piece of information we can obtain from the simulation for the [bmim][PF_6_] IL/TEGDME mixtures is to quantify the changes in the interaction between the components such as cation–anion, cation–cation, anion–anion, cation-solvent, and anion-solvent. In this section, we try to understand the microsolvation of ILs with TEGDME molecules using radial distribution functions (RDFs) to calculate the average distance between the reference component and its nearest neighbor (center of mass). The RDFs that quantify the spatial correlation between a specific atom and other similar or dissimilar atoms around it describe the short-range ordering characteristics of materials and can reflect the microstructure of the [bmim][PF_6_] IL/TEGDME mixtures, as well as is helpful to the concretization of the microscopic ionic structure in IL/TEGDME mixture. Here, it is further investigated that the influence of adding TEGDME alters the IL molar fraction on the interionic, intermolecular, and ion-molecular structural correlations based on RDFs as provided in Figure 3, Figure 4, Figure 5, Figure 6, Figure 7 and Figure 8, which presents the site−site RDFs of cation−cation, cation−anion, anion−anion, cation−TEGDME, anion−TEGDME, and TEGDME−TEGDME pairs.

The microscopic structure of the mixtures was carried out at 300 K by means of all-atom molecular dynamics (MD) simulations using compound center of mass the radial distribution functions (RDFs) for selected molecular pairs. Regarding the (O_8_–P) RDFs for anion-TEGDME pairs, Figure 3a is characterized by one peak with maxima at roughly 5.6 Å. By compared the peak around 5.6 Å of the (O_8_–P) RDFs with the concentration range from 0.1 to 0.8, the intensity is relatively higher for the concentrated TEGDME system (X_TEG_ = 0.8) compared with that of the other composition mixtures. The height of the (O_8_–P) peak decreased significantly with the decrease in the mole fraction of TEGDME from 0.70 to 0.40. When the mole fraction of TEGDME decreased from 0.40 to 0.10, the decreasing trend of TEGDME concentration gradually slowed down. Considering that the structural correlation between TEGDME and cation neighbors is mainly characterized by the strength of peak intensity, the change of RDFs of anion-TEGDME RDFs is analyzed as a function of mixture composition, Figure 3b. There are three closed peaks near 3.6, 4.5, and 6.2 Å in TEGDME–cation RDFs and the broad weak peaks with the maximum intensity of 9–12 Å. It can be concluded from Figure 3a,b that the molecular ion interactions are affected by the composition of the mixture and are gradually enhanced by the addition of TEGDME. When the ILs and TEGDME are mixed, the position and number of peaks remain unchanged in the whole composition range, which indicates the trend of the studied ions to self-associate in the study mixture with discarded isolated ions.

As shown in Figure 3a, the (O_8_–P) RDFs represent the structural correlation of anion-TEGDME neighbors, showing a peak value around 5.6 Å. Compared with the mixture of other components, the strength of peak value around 5.6 Å for the concentrated TEGDME system (X_TEG_ = 0.8) is relatively higher. When the content of TEGDME decreased from the molar fraction of 0.70 to 0.40, the peak height of (O_8_–P) decreased significantly, and such a trend occurred slightly for more dilutions. In Figure 3b, the RDFs representing the structure correlation of TEGDME–cation neighbors indicate that the enhancement of peak intensity after the addition of TEGDME is similar to that of the anion-TEGDME RDF case. The results show that there are three peaks near 3.5, 4.6, and 6.2 Å for TEGDME–cation RDFs and a broader weak peak with maximum intensity around 9–12 Å. Similar patterns can be observed in the cation–anion RDFs (Figure 3b). These RDF peaks of TEGDME–cation and cation–anion indicate different positions where a TEGDME molecule or [PF_6_]^−^ can interact with the imidazole ring of the cation. We also detected these areas by SDF analysis, as shown in Figure 8. From Figure 3a,b, we can conclude that the molecular ion interaction is affected by the composition of the mixture, which is gradually enhanced by the addition of TEGDME.

The self-aggregation of the TEGDME solvent was analyzed with the (O_8_–O_8_) RDF reported in Figure 4a, which represents the structural correlation between adjacent TEGDME molecules in the solutions. When mixing ILs with DEGMME, the position and number of peaks remained unchanged in the whole composition range, which indicated that the species studied had a tendency of self-association in the studied mixture, discarding the presence of isolated ions. (O_8_–O_8_) RDF showed the characteristic first and narrow peak near 5.5 Å, which corresponded to self-association with a significant reduction in intensity upon an increase in TEGDME mole fraction from 0.1 to 0.6, and this trend occurred slightly in more diluents until the intensity of the first (O_8_–O_8_) RDFS peak of X_TEG_ was raised up to 0.70 and 0.80. The large non-linearity of the evolution of peak intensities and weaker TEGDME–TEGDME correlations at higher contents of TEGDME molecules is the origin of macroscopic thermodynamic deviations from ideality. This (O_8_–O_8_) RDF pattern shows that there is no effective aggregation between TEGDME molecules in the studied systems, which is consistent with the experimental observation [17].

In all investigated RTILs and TEGDME mixtures, the cation–anion distance correlations were significantly stronger than all other correlations. The (ring-P) RDFs between the geometrical ring center of the [bmim]+ cation and the [PF_6_]^−^ anion were calculated as shown in Figure 4b. Although [PF_6_]^−^ preferentially interacts with TEGDME molecules present in the mixture, interaction also occurs between [PF_6_]^-^ anions and imidazolium cations, as shown by the RDF depicted in Figure 4b. Based on the cation–anion RDFs (Figure 4b), the locations of the coordination layers of cation–anion pairs representing by the (ring-P) RDFs are not changed with adding TEGDME molecules, and for the mixtures with TEGDME content from 0.7 to 0.1, the cation–anion RDFs show a distinct first shell peak suggesting the presence rising of strong interactions between the [PF_6_]^−^ species and the imidazolium ring. As the concentration of TEGDME increases to X_TEG_ = 0.70, the nearest cations and anions tend to cooperate better with themselves and form neutral ion pairs, which are stabilized by TEGDME molecules. With the mixture increase to the high level of TEGDME content, X_TEG_ = 0.80, it has the strongest first and second peaks of the (ring-P) RDF at 4.8 and 6.3 Å, but there is not the third (ring-P) peak at this mixture as seen in other more concentrated IL mixtures. Contrary to the effect of mixture composition on the heights of the first and second peaks of (ring-P) RDF, the height of the third broad weak peak decreases gradually with the addition of TEGDME at larger distances around 9–13 Å. The variation of the third broad weak peak with X_TEG_ more than 0.70 at distances around 9–13 Å is evidence of the strong dissociation of large ionic clusters in diluted IL. This is also shown in the corresponding snapshot of the simulation box in Figure 2, which corresponds to the conclusion of Section 3.3 that diluting the IL mixture will benefit the improvement of the dynamical properties.

Figure 5a shows the (ring–ring) and (P–P) RDFs representing the structural correlation between the nearest neighbors of cation–cation and anion–anion, respectively, with adding TEGDME to [bmim][PF_6_] IL systems. From Figure 5, It can be shown that the first peaks of both the (ring–ring) and (P–P) RDFs are broadened and slightly shifted to the larger distances with the increasing of TEGDME cosolvent, and the second peaks are disappeared for the deepen diluted mixture with X_TEG_ = 0.70, and especially the (ring–ring) RDF shows no cation–cation structural correlation at X_TEG_ = 0.80. Based on the cation–anion RDFs (Figure 5a) and anion–anion RDFs (Figure 5b), it can be shown that the increase in TEGDME content leads to the weakening of cation–cation and anion–anion association complexes, and as a result in the transformation of species into isolated forms. In other words, the number of free ion pairs will increase gradually with the addition of TEGDME, while it will be decreased for the number of large ion clusters composed of many associated ion pairs, which are strongly associated by polar and nonpolar domains. These conclusions are consistent with the experimental results [16].

The inter/intramolecular interactions in neat TEGDME molecules are mainly van der Waals force, which is weaker than the [bmim][PF_6_] IL systems containing strong hydrogen-bonding or electrostatic interactions. When the neat TEGDME mixes with [bmim][PF_6_], the hydrogen-bonding interactions between the oxygen of ether bond in TEGDME and the imidazolium ring proton as well as the interactions between ions of ionic liquid cause a more compact structure, which results in the slower rotation of C153 and the higher microviscosity compared to the neat TEGDME. On the other hand, increasing of TEGDME into [bmim][PF_6_] causes the weakening of cation–cation and anion–anion associations so that the species are switched to the isolated form, which attributes to the improvement of dynamical properties at diluted IL mixtures. Accordingly, it can be said that by adding TEGDME, the number of free ion pairs should be more than that of large ionic clusters, consisting of many associated ion pairs, which are constructed by the strong association of polar and nonpolar domains. These results are consistent with the findings of the experiment [16].

#### 3.2.3. The Hydrogen Bonds in the IL/TEGDME Mixture

As discussed above, in pure ionic liquids, the electrostatic interaction between ionic species is the dominating interaction, which directly leads to the close packing of ions in the ionic structure. In addition, the thermodynamic, transport, and other properties of ionic liquids are also strongly dependent on the electrostatic interactions between ionic species [62]. In the IL/TEGDME mixture, not only the electrostatic interaction of the atomic center but also the hydrogen bond interaction plays an important role in the related properties of ionic liquids. Here, we investigate the formation of hydrogen bonds between ionic species and TEGDME molecules in IL/TEGDME mixtures with varied TEGDME mole fractions.

As shown in Figure 6, the H-bonds are analyzed by examining the site-site RDFs between the hydrogen atoms of [bmim]+ cations and the negatively charged fluorine atoms of [BF_6_]^−^ anions. The first maximum peak is located at 0.254 nm for the C_3_-H_5_…F pair, 0.255 nm for the C_5_-H…F pair, 0.256 nm for the C_7_-H… F pair, 0.265 nm for the C_9_-H…F pair, and 0.490 nm for the C_22_-H…F pair. Based on these RDFs, the first peak intensities of (X-F) RDFs were observed as the following trend: X = H_4_ > H_6_ > H_8_ > H_11_. It can be seen that for (H*_n_*–F, *n* = 4, 6, 8, 11) RDF, a relatively strong peak at around 2.5 Å is found, while for (H_25_–F) RDF, the first peak is observed at ~4.9 Å with a lower intensity. This provides one evidence of the strong hydrogen bond between the head group of the cation (imidazolium ring) and [PF_6_]^−^, which seems to be one of the reasons for the formation of large ionic clusters and the increase in viscosity in the IL environment.

As shown in Figure 6b, the hydrogen bonding representing structural correlations between [bmim][PF_6_] IL and TEGDME is illustrated by simulating RDFs between the hydrogen atom (H_4_) of the cations and selected oxygen atoms of the TEGDME molecules (O_2_, O_5_, O_8_, O_11_, O_14_) for the mixture with X_TEG_ = 0.6 at 300 K. Based on these (H_4_–O*_n_*, *n* = 2, 5, 8, 11, 14) RDFs (Figure 6b), the first peak intensities of (H_4_–O*_n_*, *n* = 2, 5, 8, 11, 14) RDFs were observed as the following trend: X = O_8_ > O_5_ > O_11_ > O_2_ > O_14_. Accordingly, we can notice that there is a strong structural correlation caused by the hydrogen bond between the O_8_ atom of the TEGDME molecule and the head group of the cation.

According to the above hydrogen bond-based structural studies of [bmim][PF_6_] IL/TEGDME mixtures, the strongest structural correlations were investigated between the anion [PF_6_]^−^ and imidazolium cations that are related to the interactions of the negatively charged fluorine atoms of anions and the hydrogen atom of the imidazolium ring. A more detailed structural analysis of [bmim][PF_6_] IL/TEGDME mixtures was also obtained by calculating the RDFs between the hydrogen atom (H_4_) of the cations and selected oxygen atoms of the TEGDME molecules. It seems that the strong hydrogen bonding occurs between the anions and the head group (imidazolium ring) of the cations as well as the TEGDME molecules and the head group (imidazolium ring) of the cations.

The relative stability of ion-pair configuration is determined by electrostatic attraction and hydrogen-bonding interaction between the ions of opposite charge. It is found that (H_4_-O_8_) RDF, which is used to describe the strengths of H-bonds between the IL anion and the TEGDME, linearly decreases and is well correlated with the mole fraction of TEGDME in the mixtures, as shown in Figure 7a. The H-bond details between the cations and anions are also further shown in Figure 7b by analyzing the (H_4_-F) RDFs under the different TEGDME mixtures. It is found that, with the addition of TEGDME, the strengths of H-bonds between the fluorine atoms and the hydrogen atoms on the pyridine rings increase first and reach a maximum when the TEGDME mole fraction reaches about 0.6, then decreases with further addition of TEGDME.

#### 3.2.4. Spatial Distribution Functions

In order to obtain a three-dimensional picture of the structural distribution of ions and cosolvent molecules in [bmim][PF_6_] IL/TEGDME binary mixtures at different compositions and to better understand the polarity impact on solution structure, we next obtain the composition-dependent spatial distribution functions (SDFs) by selecting the center of masses of [PF_6_]^−^ (red density clouds) around [bmim]+ and TEGDME (yellow density clouds) around [bmim]+ as the reference cation via the VMD software. As shown in Figure 8, the SDF of anions around cations and the TEGDME around cations in three systems with different TEGDME mole fractions at 300 K and 1atm were derived. Considering that the spatial density in the graph is relative, we adjust the isovalue to an improper value to clearly display the figure of the SDFs could.

Combined with the above analysis of the RDFs and SDFs, it can be shown that the anions [BF_6_]^−^ are mainly distributed around the cationic pyridine ring, which is due to the strong electrostatic interactions and hydrogen bond between H*_n_* (*n* = 4, 6, 8, 10, 11, 12) on the rings and F on [BF_6_]^−^. However, for the TEGDME, it is distributed not only around the cationic pyridine ring due to the hydrogen bond between H*_n_* (*n* = 4, 6, 8, 10, 11, 12) on the rings and O*_n_* (*n* = 2, 5, 8, 11, 14) on the TEGDME but also around the tail of the alkyl chain for the VDW interaction. As observed by comparing first column panels of the SDFs at 300 K in Figure 8, it can be found that more SDFs of the anions around the pyridine ring are shown with the increase in the TEGDME molar fraction. By comparing the second column panels of the SDFs in Figure 8, we can see that the probabilities of the TEGDME molecules around the pyridine ring increase with the rise of the TEGDME molar fraction. A similar phenomenon could be observed for the TEGDME around the tail of the alkyl chain, which increases with the rise of the TEGDME molar fraction.

Based on the RDFs and SDFs results, it can be concluded that the nonpolar cosolvent TEGDME without the presence of a hydroxyl group could efficiently affect the nonpolar domains and the polar domains of [bmim][PF_6_]. As TEGDME molecules have a certain length nonpolar chain, the TEGDME has a great affinity to the imidazolium ring of the cation belonging to the polar domains through the hydrogen-bonding effect with the ethers of TEGDME, which may act as a hydrogen bond acceptor, and TEGDME could also incline to the hexyl side chain of the cation belonging to the nonpolar domains through van der Waals interactions. Thus, in the [bmim][PF_6_] IL/TEGDME binary mixtures, the TEGDME molecules can reside as a bridge between the polar and nonpolar domains in agreement with previous research [17]. When the TEGDME mole fraction increase from 0 to 0.5, the TEGDME molecular chain gradually makes the incompact domain more and more dense for the packing and stacking effects of the TEGDME molecular chain. When the mole fraction of TEGDME is further increased from 0.5 to 1, the heterogeneous structure of the mixture is gradually destroyed due to the more TEGDME enrichment stage in the mixture.

### 3.3. Dynamical and Transport Properties of [bmim][PF_6_]/TEGDME Mixtures

In this section, the dynamical properties, microstructure, and thermodynamics, such as the rotational autocorrelation functions (RACFs), the mean square displacements (MSDs) and for the center of mass of ions and TEGDME molecules, and the relative diffusivities as a function of X_TEG_, is calculated by extensive molecular dynamics simulations at 300 and 353 K, so as to have a deeper understanding of the microscopic motion and dynamic behavior of different species. Furthermore, by using the Nernst–Einstein equation, the ionic conductivity of [bmim][PF_6_]/TEGDME solutions was also estimated from the ionic self-diffusion coefficients to found the influence of adding TEGDME as a cosolvent on the transport properties of [bmim][PF_6_] IL, especially for electrochemical applications.

#### 3.3.1. Rotational Dynamics of C153 in [bmim][PF_6_] and TEGDME Mixtures

Since the coumarin 153 (C153) is considered to be an ideal probe related to nonspecific solute-solvent interactions in many RTIL systems, we choose the C153 to study how its rotational dynamics are affected by the changes of microstructure in different structural domains of [bmim][PF_6_] before and after adding cosolvent TEGDME at different fractions. In this work, the C153 rotational correlation function was calculated in [bmim][PF_6_] and [bmim][PF_6_]/TEGDME mixtures across all TEGDME mole fractions [63]. The relaxation times were fitted from the correlation function curve to the following function:

(2) $$r(t)=\sum_{i=1}^{2}a_{i}e^{-t/\tau_{i}}$$

where *τ*_1_ and *τ*_2_ represent the relaxation times of C153 and ai is a proportion parameter.

The rotational autocorrelation function (RACF) of C153 in pure [bmim][PF6] and [bmim] [PF_6_]/TEGDME mixtures across all mole fractions are listed in Table 2, which reflects the concentration dependence of the rate of reorientation or rotational motion rate of C153. As shown in Figure 9 and Table 2, two different time constants are obtained by fitting the decay of the correlation function curve of C153 in neat [bmim][PF_6_] at 300 K and 353 K, respectively. It is found that, with the addition of TEGDME, the value of the fast rotation time constant (*τ*_1_) in [bmim][PF_6_] and TEGDME mixtures increases first and reaches a maximum when the TEGDME mole fraction reaches about 0.5, then decreases with further addition of TEGDME. Regarding the values of the slow time constant (*τ*_2_) obtained from the C153 rotational dynamics measurements, it is found that *τ*_2_ linearly decreases and is well correlated with the mole fraction of TEGDME in the mixtures, as shown in Figure 9a. The trend for the values of the fast rotation time constant (*τ*_1_) and the slow time constant (*τ*_2_) found in the [bmim][PF_6_] and TEGDME mixtures at 300 K is consistent with those observed in our previous experiments [16]. Because the slow decay of the rotational dynamics of C153 is related to the microviscosity of the compact domain of the mixture, the interaction between the two pure components may destroy the structure of the compact domain, which is composed of imidazole ring cations and anions, resulting in the decrease in the microviscosity. These conclusions are consistent with the experimental results, which study the rotational dynamics of C153 by using the time-resolved fluorescence anisotropy spectroscopy method [16].

#### 3.3.2. Diffusion Dynamics of Ions in [bmim][PF_6_] and TEGDME Mixtures

Absolute self-diffusion coefficients as a function of mixture composition. For a liquid or mixture in the conditions of thermodynamic equilibrium, the self-diffusion coefficient is defined as the translational motion of the liquid molecule caused by thermal agitation. From the molecular point of view, the self-diffusivity of liquid molecules gives a detailed microscopic description of single-particle motion. There are many factors affecting the diffusion coefficients of anions and cations in ionic liquids. Some of these factors are the relative cationic and anionic size, geometric shape, mass, surface electrical charge density on the ions, the intensity of local van der Waals interaction between cations and anions, and the intensity of Coulomb interaction between cations and anions, which depend on the localization of charge on ions.

In this work, the method of describing the dynamics of particle diffusion in dense fluid is explored to obtain the self-diffusion coefficient by using Einstein relation and the mean square displacement (MSD) by using the Einstein relation. The MSD is defined by:

(3) $$\mathrm{MSD}=\frac{1}{N}\langle \sum_{i=1}^{N}{\left|{\vec{r}}_{i}\left(t\right)-\vec{r_{i}}\left(0\right)\right|}^{2}\rangle =\Delta {\left|r\left(t\right)\right|}^{2}$$

where ${\vec{r}}_{i}\left(t\right)$ is the location of the center of mass of ion *i* at time *t*.

And accordingly, the self-diffusion coefficient can be calculated from the long-time limit of the MSD using the well-known Einstein relation. The calculated self-diffusion coefficients of the ions of [bmim][PF_6_] IL and TEGDME molecules in the case of pure and the various molar fractions of [bmim][PF_6_] IL/TEGDME binary mixtures are shown in Table 3, which were calculated at 300 and 353 K from the MD trajectories using the Einstein relation:

(4) $$D=\frac{1}{6}\underset{t\to \infty }{\lim\frac{d}{\mathrm{dt}}}\langle {\left[{\vec{r}}_{i}\left(t\right)-\vec{r_{i}}\left(0\right)\right]}^{2}\rangle$$

where the quantity $\langle {\left[{\vec{r}}_{i}\left(t\right)-\vec{r_{i}}\left(0\right)\right]}^{2}\rangle$ is the ensemble-averaged MSD of the molecules, and *r*_i_ is the vector coordinate of the center of mass of ion *i*.

It should be noted that the simulation time is long enough for Equation (4) to hold because ILs exhibit very slow diffusion behavior. In addition to the starting region of MSD, the ending region should be abandoned for Equation (4) due to the inaccuracy caused by insufficient sampling. Therefore, in this work, we calculate the MSD of the ions by using the trajectories of relatively larger than 10 ns. The starting point and time domain of MSDs’ linear fitting are different from each other in the literature. It is reported that the error for estimating the self-diffusion coefficient (SDCs) by using MSD calculations is the shorter the run length used for fitting, the greater will be the overestimation of the self-diffusion coefficient, and fitting should avoid the initial and final regions [29]. Here, the slope is estimated by linear regression under the condition that trajectories were dumped for 1 ns and the range 200–600 ps. The calculated self-diffusion coefficients of the three components [bmim]+, [BF_6_]^−^ and TEGDME, represented by D+, D− and D_TEG_, respectively, are presented in Table 3 for the full composition ranges at 300 and 353 K with 1 atm. As is shown in Table 3 and Figure 10, these results show a non-linear behavior of the self-diffusion coefficients for the three components all and increase with the addition of TEGDME molecules.

In pure [bmim][PF_6_] IL system (X_TEG_ = 0.0) at 300 K, the computed values of D+ and D− are 2.97 and 1.04 (×10^−12^ m^2^·s^−1^) respectively, that are in the same order of magnitude with the available experimental values measured by NMR, which are 7.1 and 5.4 (×10^−12^ m^2^·s^−1^) at 300 K [35]. With the addition of TEGDME to X_TEG_ = 0.40 and 0.60, the value of D+ is 7.69 and 18.5 (×10^−12^ m^2^·s^−1^), respectively at 300 K. As at the same compositions, the computed value of D− is 5.45 and 19.8 (×10^−12^ m^2^·s^−1^) at 300 K, respectively. So, compared X_TEG_ = 0.40 and 0.60 with pure [bmim][PF_6_] IL system (X_TEG_ = 0.0) at 300 K, D+ rises up to ~3 and 7 times and D− increases to ~5 and 19 times, respectively. With more increasing the TEGDME molecular fraction (X_TEG_ = 0.70 and 0.80), a very significant increment is observed both in the D+ and D− diffusion values, among which D+ and D− are ~17 and 52 times of magnitude higher than the pure IL at X_TEG_ = 0.80.

From Table 3, we can see, for the pure TEGDME system (X_TEG_ = 1.0) at 300 K, the computed D_TEG_ is 230.7 (×10^−12^ m^2^·s^−1^). When the TEGDME component further decreases as X_TEG_ is down to 0.80, the value of D_TEG_ decreases to 91.7 (×10^−12^ m^2^·s^−1^), so that D_TEG_ decreases to ~2.5 times lower than that of the pure TEGDME. As X_TEG_ is down to 0.60, the value of D_TEG_ decreases to 36.6 (×10^−12^ m^2^·s^−1^), so that D_TEG_ decreases to ~6.3 times. As the TEGDME content further decreases (X_TEG_= 0.40 and 0.10), a very significant decay is observed in the diffusion coefficient values of the TEGDME component, 17.1 and 6.8 (×10^−12^ m^2^·s^−1^), and then D_TEG_ is ~13.5 and 34 times of magnitude lower than that of the pure TEGDME simulated system, respectively.

The simulations for [bmim][PF_6_] IL/TEGDME mixture at 353 K are also given. As compared from Figure 10 and Table 3, a similar trend of the self-diffusion coefficients for mixture at 353 K is acquired in the composition dependence of D_TEG_. The relative computed D_TEG_ with the composition of mixtures at 353 K is a little higher than those of at 300 K. As shown in Table 3, the calculated diffusion coefficients of the free ions D+ and D- for pure [bmim][PF6] IL system (X_TEG_ = 0.0) were 2.97 and 1.04 (×10^−12^ m^2^·s^−1^) at 300 K increasing up to 8.81 and 5.30 (×10^−12^ m^2^·s^−1^) at 353 K, respectively. As the IL content further increases (X_TEG_ = 0.5), the computed value of D+ and D- is 46.1 and 46.5 (×10^−12^ m^2^·s^−1^), respectively at 353 K. So, compared the same compositions (X_TEG_ = 0.50) at 300 K, D+ rises up to ~4.64 times and D− increases to 4.43 times, respectively. With more increasing the TEGDME molecular fraction (X_TEG_ = 0.80), an increment is observed both in the D+ and D- diffusion values when the temperature is raised to 353 K, among which D+ and D- are 145.9 and 168.9 (×10^−12^ m^2^·s^−1^) higher than 49.6 and 53.6 (×10^−12^ m^2^·s^−1^) simulated at 300 K.

As is analyzed above for the transport dynamics of different components in the mixture within the full composition ranges, it can be shown that with the increase in temperature and the dilution of IL and TEGDME, the self-diffusion coefficients of ions and TEGDME molecules increase significantly, which may be due to the changes in the cation–cation, anion–anion, TEGDME–TEGDME, cation–anion, cation-TEGDME, and anion-TEGDME neighboring structural correlations. Three regions can be inferred from the composition evolution of self-diffusion coefficients D in the mixture: (I) in the region of above 0.70 TEGDME mole fraction, D decreases sharply compared with pure TEGDME; (II) 0.50–0.70 mole fraction of TEGDME, which seems to be a transition region; And (III) regions with TEGDME mole fraction less than 0.50 seem to be dominated by IL. In these three regions, the ordering of D is D+(cation) > D− (anion) and D_teg_.

These results can be explained by the fact that the fluids dominated by TEGDME in region 1 have isolated ion pairs solvated by TEGDME molecules (Figure 10). With the increase in TEGDME concentration, a large number of TEGDME cosolvent molecules are inserted into the interface region of the polar network and nonpolar domain, and their movement speed is obviously faster than that of ions. Therefore, the dissociation of ion clusters leads to a sudden increase in ion mobility. It further promotes the dissociation of aggregated ion pairs and leads to higher ion mobility. Transition region 2, where D_TEG_ decreases compared with the value in region 1, resulting in a value close to that of ions D+ and D−, indicating that the diffusion of TEGDME molecules is highly correlated with ions, so the aggregation of ions should increase, leading to larger clusters, and hydrogen bonding to TEGDME molecules (Figure 11). Region 3 corresponds to a fluid dominated by large anion-cation clusters due to van der Waals and Coulomb interaction, which involves polar networks and nonpolar aggregation domains, and TEGDME is dispersed in void spaces, so it results in transmission-blocking and has very low mobility.

By comparing the composition of cation, anion, and dimethyl ether in the whole composition range and the temperature dependence of the self-diffusion coefficient, the results show that with the increase in temperature and the dilution of IL and TEGDME, the self-diffusion coefficient of ions and TEGDME increases significantly, which may be due to the variations in the cation–cation, anion–anion, TEGDME–TEGDME, cation–anion, cation-TEGDME, and anion-TEGDME neighboring structural correlations. The various interactions among the cations, the anions, and the TEGDME molecule, including Coulomb interactions, hydrogen bonding, van der Waals interactions, and dispersion interactions, collectively result in the complex behavior of the transport dynamics in mixtures.

Relative self-diffusion coefficients as a function of mixture composition. Recently, an abnormal phenomenon of ionic diffusion coefficient has been found in RTIL-solvent system; that is, by heating and/or diluting with molecular solvent, the unexpected low diffusion rate of smaller anions in pure RTIL can be improved more effectively than that of larger cations. In other words, a reversal is expected under certain dilution/heating conditions. According to various experimental observations, the reversal point of relative ionic diffusivity was found near the equimolar composition in the [bmim] [PF_6_]-based system and did not rely on the solvent. At this point, the ion diffusion mechanism has changed obviously. At low RTIL content, the diffusion rate of anions is faster than that of cations as anticipated from their relative sizes, while at high concentration and pure RTIL, the diffusion rate of anions is slower. In order to explain the rather universal behavior of RTIL-solvent mixtures, it is necessary to study the diffusion mechanism separately.

The solvent-independent inversion of ionic diffusivities motivates us to estimate the relative diffusivity ratios of D_TEG_/D+, D_TEG_/D−, and D−/D+ as a function of the TEGDME molar fraction at 300 K and 353 K (Figure 11). An important observation in Figure 11 is that the self-diffusion of [PF_6_]^−^ rather than [bmim]+ follows an inverse trend in both concentrated and diluted solutions. In neat IL and concentrated IL mixtures (X_TEG_ ≤ 0.50), D+ is larger, which means that the self-diffusion of cations is higher than that of corresponding anions, but for diluted IL, that is when X_TEG_ > 0.50, the opposite expected trend is found. As is shown in Figure 11, D− constitutes ~110%–120% of the D+ at X_TEG_ > 0.50. In other words, anions [PF_6_]^−^ diffuse faster than cations [bmim]+ in the diluted IL solutions by TEGDME, whereas at the concentrated [bmim][PF6] IL solutions, the opposite trend is observed.

There are different explanations for the abnormal phenomenon of the ionic diffusion coefficient in the RTIL-solvent mixture system. One explanation of this phenomenon was proposed and later claimed the hypothesis of hyper-anion prevalence (HAP) by Chen et al. [64]. This hyper-anion prevalence (HAP) hypothesis implies that in pure RTIL and concentrated solutions, there exist not only neutral ion aggregates but also predominantly singly charged associates. This hyper-anion prevalence (HAP) hypothesis implies that in pure RTIL and concentrated solutions, there exist not only neutral ion aggregates but also predominantly singly charged associates. According to this hypothesis, these charged aggregates should preferably be enriched with anions. In other words, aggregates with a positive charge are usually smaller than those with a negative excess charge. Therefore, if this HAP hypothesis is followed, anions are mainly distributed in larger aggregates, which diffuse slower than the smaller cation enriched aggregates. Accordingly, in pure RTILs and concentrated solutions, the natural supramolecular network structure of pure RTILs was maintained, D+ > D−, despite the opposite prediction from ion size. After more dilution and/or heating, the aggregates become smaller and smaller until complete dissociation, and individual ions are typical diffusive species. At this phase, the expected relative order of diffusion coefficients is found. This method is also used to explain similar observations on aqueous systems.

A complementary explanation can be presented here for the diffusion mechanism of anions with the help of our structural analysis reported above in Section 3.2. In the presence of TEGDME molecules, the interaction between species has changed significantly, which has a profound impact on the transport properties. In the pure and concentrated [bmim][PF_6_] IL, the cations and anions are bound together. The addition of TEGDME leads to the simultaneous dissociation of ionic aggregations, and there appears a correlation between ions and TEGDME molecules. It seems that in the TEGDME-rich region that diluted IL solution with X_TEG_ > 0.50, in addition to the separation of ionic aggregations, interactions of the nearest TEGDME–cation neighbors are much strengthened compared to those of TEGDME-anion neighbors so that the number of isolated anions is more than that of isolated cations at this condition. Therefore, a significant enhancement is observed in the D−/D+ ratio and in the relative dynamics of [PF_6_]^−^ anions in diluted [bmim][PF_6_] IL (Figure 11). The results of structural analysis (see Section 3.2, Figure 2, Figure 3, Figure 4, Figure 5, Figure 6, Figure 7 and Figure 8) complementary confirm our dynamical observations and the above discussion.

## 4. Conclusions

In this study, tetraethylene glycol dimethyl ether (TEGDME) is selected as a cosolvent with imidazolium-based ionic liquid 1-butyl-3-methylimidazolium hexafluorophosphate [bmim][PF_6_] to study the interactions and properties of the polar and nonpolar domains in such a hybrid binary system. The structural, dynamical, transport, and thermodynamic properties for the binary mixtures of the ([bmim][PF_6_]) IL with TEGDME solution have been calculated over the whole concentration range at *T* = 300−353 K and atmospheric pressure *p* = 1 atm. At the same time, consider the complex cation–cation, anion–anion, TEGDME–TEGDME, cation–anion, cation-TEGDME interactions, anion-TEGDME, and diverse microstructures that existed in the [bmim][PF_6_] IL/TEGDME binary mixtures, through calculation of the RDFs and SDFs, the mechanism of complex behavior of transport mechanics in mixtures is studied, which mainly focus on the various interactions among the cations, the anions and the TEGDME molecule, including Coulomb interactions, hydrogen bonding, van der Waals interactions, and dispersion interactions. It is found that the self-diffusion coefficients for [bmim]^+^, [PF_6_]^−^, and TEGDME molecule in [bmim][PF_6_]/TEGDME mixtures will increase gradually with the addition of TEGDME, while it will be decrease for the D_Teg_/D+ and D_Teg_/D− ratio, which are strongly associated by ionic aggregations. MD simulations also show that the TEGDME molecules reside as a bridge between the polar and nonpolar domains in the binary mixtures, which has a great affinity to the imidazolium ring of the cation belonging to the polar domains through the hydrogen-bonding effect with the ethers of TEGDME that may act as a hydrogen bond acceptor and could also incline to the hexyl side chain of the cation belonging to the nonpolar domains through van der Waals interactions.

## Figures and Tables

**Table 1:** Data on the simulated boxes prepared for pure [bmim][PF_6_] IL and its binary systems at 300 K. Mainly include X_TEG_, total [bmim][PF_6_] ion pairs, Tetraethyleneglycol dimethyl ether(TEGDME) molecules, atoms, and sizes, and the van der Waals cutoff distances, R_Cutoff_, of the studied systems at 300 K.

x_TEG_	*N*_TEG_	*N*_ion_ Pairs	Total Atoms	Box Sizes (*X*, *Y*, *Z*) /Å	R_cutoff_/nm	Density g/cm^3^
0.00	-	400	12,823	52.67 × 52.67 × 50.01	1.0	1.351
0.10	40	360	12,143	53.33 × 53.33 × 49.06	1.0	1.316
0.40	160	240	10,103	51.59 × 51.59 × 51.10	1.0	1.213
0.60	240	160	8743	51.03 × 51.03 × 51.56	1.0	1.146
0.70	280	120	8063	51.31 × 51.31 × 51.35	1.0	1.113
0.80	320	80	7383	51.26 × 51.26 × 51.07	1.0	1.078
1.00	400	-	6023	51.94 × 51.94 × 50.48	1.0	1.012

**Table 2:** Experimental and simulated rotational relaxation parameters for C153 in [bmim][PF_6_] and TEGDME mixtures at 300 and 353 K under 1 atm.

TEGDME Mole Fraction	Experiment		MD Simulation			
	*τ*_1_ (ps)	*τ*_2_ (ps)	*τ*_1_ (ps)		*τ*_2_ (ps)	
	300 K	300 K	300 K	353 K	300 K	353 K
0.0	0.32	4.90	0.41	0.26	8.67	4.97
0.1	0.39	4.45	0.45	0.37	7.85	4.56
0.2	0.48	3.90	0.52	0.41	6.56	4.01
0.3	0.65	3.61	0.78	0.52	5.97	3.51
0.4	0.73	2.88	0.81	0.61	4.89	2.71
0.5	0.77	2.64	0.85	0.56	4.31	2.59
0.6	0.76	2.13	0.83	0.52	3.56	2.07
0.7	0.74	1.63	0.79	0.50	2.21	1.52
0.8	0.70	1.19	0.77	0.47	1.67	1.21
0.9	0.63	0.63	0.73	0.43	0.81	0.57
1.0	0.46	0.46	0.71	0.39	0.71	0.39

**Table 3:** The self-diffusion coefficients (D in 10^−12^ m^2^/s) of ions and TEGDME molecules obtained from the slope of MSD plots in various TEGDME molar fractions of the studied [bmim][PF_6_]/TEGDME systems at 300 and 353 K.

System (X_teg_)	300 K				353 K			
	D+	D−	D_teg_	D_sys_	D+	D−	D_teg_	D_sys_
0	2.97	1.04	--	1.94	8.81	5.30		7.02
0.1	3.49	1.39	6.82	2.73	12.1	7.67	22.2	10.8
0.2	4.21	2.26	9.04	4.16	17.8	13.5	42.1	19.9
0.3	5.86	3.03	12.6	6.45	27.1	20.7	51.9	30.9
0.4	7.69	5.45	17.1	10.5	34.6	30.4	60.6	42.0
0.5	9.92	10.5	24.6	17.6	46.1	46.5	80.5	61.3
0.6	18.5	19.8	36.6	28.6	72.6	75.5	119.7	98.4
0.7	29.7	33.1	57.3	48.1	110.2	115.1	179.4	156.2
0.8	49.6	53.6	91.7	82.0	145.9	168.9	248.2	225.8
0.9	84.8	99.8	177.7	166.8	201.1	238.9	373.5	355.3
1.0	-	-	230.7	230.9			543.7	541.7

## Data Availability

The raw data of this paper are fully accessible upon request to the authors.
